# Interventions to Foster Mental Health and Reintegration in Individuals Who Are Unemployed: Systematic Review

**DOI:** 10.2196/65698

**Published:** 2025-05-05

**Authors:** Sophia Helen Adam, Florian Junne, Svenja Schlachter, Miriam Mehler, Harald Gündel, Nicolas Rüsch, Jörn von Wietersheim, Katrin Elisabeth Giel, Stephan Zipfel, Rebecca Erschens

**Affiliations:** 1 Department of Psychosomatic Medicine and Psychotherapy Internal Medicine University Medical Hospital Tuebingen Tuebigen Germany; 2 Department of Psychosomatic Medicine and Psychotherapy Otto-von-Guericke University Magdeburg Magdeburg Germany; 3 Department of Psychosomatic Medicine and Psychotherapy Ulm University Medical Center Ulm Germany; 4 Department of Psychiatry II Ulm University and BKH Guenzburg Ulm Germany

**Keywords:** cognitive behavioral therapy, depression, job loss, mental health, re-employment, unemployment

## Abstract

**Background:**

Unemployment is a risk factor for the development and perpetuation of psychological distress. Finding support for affected individuals can be particularly challenging, which causes a vicious cycle of psychological distress and unemployment.

**Objective:**

The aim of this systematic review is to assess and summarize existing evidence regarding interventions that address both mental health and re-employment, emphasizing accessibility through community or social care structures.

**Methods:**

A systematic literature search using PubMed and EBSCOhost and an additional search using reference list screening were conducted according to the PRISMA (Preferred Reporting Items for Systematic Reviews and Meta-Analyses) guidelines. In order to identify interventions for the mental health and re-employment of individuals experiencing psychological distress and unemployment, an inclusion process according to the PICO (population, intervention, comparison, and outcome) scheme and the study design was applied. Title and abstract screening and full-text screening for eligibility were performed independently by 2 reviewers. Quality assessments using the Cochrane Risk of Bias Tools for randomized and nonrandomized trials were conducted by 2 independent reviewers.

**Results:**

The initial systematic search yielded 4442 results, and 15 articles were additionally identified via reference list screening. Eventually, 74 articles were subjected to a thorough evaluation process by 2 independent reviewers. The interrater reliability was determined to be good, with a Cohen κ score of 0.770. After a multistep extraction process, 17 studies remained for inclusion, with each focusing on the improvement of mental health, re-employment, or both outcomes. A heterogeneous pattern of results emerged, with most interventions showing improvement in either mental health or re-employment. Most studies were assessed as having a moderate (n=7) or high (n=9) risk of bias.

**Conclusions:**

The results of the systematic research indicate that low-threshold services in close cooperation with institutions and exchange with other supportive stakeholders should be fostered. Derivable overarching themes and intervention content for integrative support measures can serve as guidelines for future interventions.

**Trial Registration:**

PROSPERO CRD42022378490; https://www.crd.york.ac.uk/PROSPERO/view/CRD42022378490

## Introduction

### Background

The salutogenic and protective effects of employment have been repeatedly confirmed [1,2], emphasizing that employment has psychologically relevant functions that go far beyond earning money. Despite international statistics showing a steady downward trend in unemployment rates (European Union: 6.1%; United States: 5.4% [3,4]), it remains a persistent societal issue. This is due to the yet unforeseeable effects of migration and economic transformation processes, including the introduction of artificial intelligence. Furthermore, due to the prevalence of less stable employment relationships and a trend toward self-employment in modern working biographies, flexible and remote working conditions, as well as periods of unemployment, are likely to persist as common features of many working biographies.

### The Vicious Cycle of Mental Illness and Unemployment: A Glass Ceiling When Looking for Support

Unemployment is accompanied by financial distress, and there is also a well-researched negative association between mental health and unemployment [5]. Numerous studies have shown that the prevalence rates of clinically relevant mental health problems, such as symptoms of depression, anxiety disorders, and psychosomatic illnesses, are increased in the unemployed population compared with the employed population [6,7]. While the point prevalence of depression in the general population ranges between 6.38% [8] and 12.9% [9], an aggregate increased prevalence of 24% persists for individuals who are unemployed [7]. Furthermore, unemployment was shown to be a negative predictor for the effectiveness of psychotherapeutic interventions [10]. The adverse effects of unemployment are particularly severe for those experiencing additional difficulties. This often results in additional barriers to re-employment, which in turn leads to a self-reinforcing cycle [5,11].

Despite the evidence-based salutogenic effect of employment [6,12], which is further supported by growing research on individual placement and support [1,13,14] and the frequent wish for normal employment among people with mental illness [15,16], a negative correlation between the severity of mental illness and the likelihood of re-employment has been found [17].

Similarly, general subjective psychological stress can reduce the re-employment rate of newly unemployed individuals [18]. In the long run, mental disorders and associated limitations can prolong the job search process, as poor physical and mental health can negatively impact the chances of re-employment [19]. It was also shown that people with severe mental disorders, such as schizophrenia and bipolar disorder, have significantly lower levels of employment before and after being diagnosed. Hakulinen et al [20] showed that more than half of all people with a serious mental disorder had no earned income after being diagnosed. However, even if individuals do find employment, their integration can be challenging as employers hold stigmatizing attitudes and prejudices toward those with mental illnesses. For example, employers may view them as being less reliable [21]. Thus, frequently encountered challenges when working with a target group that is subjected to multiple stressors cannot be disregarded. Hagen et al [22] noted that individuals in social welfare systems face difficulties accessing treatment services. This may partly be attributed to the stigma associated with both unemployment and mental illness, leading to barriers for seeking health care and psychosocial support [23-25].

### The Need for Low-Threshold and Dual-Path Interventions

Individuals experiencing psychological distress and unemployment have an urgent need for specific interventions aimed at improving their mental health in addition to the essential goal of re-employment. Given the difficulties outlined above, employment agencies often play a crucial role in providing support, for example, by offering initial assistance, as they can provide viable access to support and initial contact with further support mechanisms. However, despite the urgent need to provide individuals experiencing psychological distress and unemployment with adequate support [26] to act preventively and to ensure long-term employment as well as reduce the risk of chronification, tailored programs for unemployed people with mental stress and illness are still rare [16,27].

Several previous reviews and meta-analyses have already addressed interventions in the context of mental illness and unemployment. Some of them have focused on health-promoting interventions for unemployed individuals in general (eg, those also including physical exercise [28-30]). Others have highlighted the effects of interventions on mental health outcomes, but they did not include re-employment as an outcome [31,32]. van Rijn et al [33] and Audhoe et al [34] investigated the effects of vocational interventions on re-employment outcomes. The results indicated that especially subpopulations at high risk of depression benefit the most from interventions [32]. However, most reviews have shown marginal to small effects on health outcomes and quality of life [28,31] and weak evidence for the effectiveness of vocational interventions on re-employment and mental health outcomes [34]. Eventually, Koopman et al [30] described the greatest benefit in combining health promotion approaches with re-employment.

While previous reviews primarily focused on either interventions targeting mental health outcomes or those aiming to improve re-employment rates, this systematic review provides a comprehensive synthesis of interventions that address both aspects simultaneously. By considering both re-employment and mental health as interconnected outcomes, this review extends prior work that has examined these domains separately. Moreover, a distinctive contribution of this review is its focus on interventions that are accessible through community or social care structures, rather than those limited to clinical or workplace settings. Thus, this systematic review provides a synthesis of available research on interventions and programs to promote the mental health and re-employment of individuals affected by mental strain and unemployment. This systematic review was conducted to investigate the following research questions:

1. What types of interventions have been implemented to promote mental health and facilitate re-employment among unemployed individuals, and what are their characteristics and outcomes?
2. How effective are these interventions in enhancing mental health outcomes and improving re-employment rates?

## Methods

### Search Strategy and Data Extraction

All data are included in this report. Data were analyzed using EndNote version 20 [35] and visualized using the risk of bias visualization (robvis) tool [36]. The procedures and results of this systematic review are in accordance with the PRISMA (Preferred Reporting Items for Systematic Reviews and Meta-Analyses) guidelines [37,38]. For the PRISMA checklist, see Multimedia Appendix 1. This study has been registered with PROSPERO (CRD42022378490). The databases PubMed and EBSCOhost (Academic Search Premier, APA Psych articles, APA PsycInfo, MEDLINE, PSYNDEX, and Business Source Premiere) were searched on October 3, 2022. For the search terms used, see Multimedia Appendix 2. Abstracts and titles were included in each of the searches. The initial search was updated on July 7, 2023. Moreover, ongoing and planned clinical trials were searched using the International Clinical Trials Registry Platform (ICTRP). The inclusion strategy was based on the PICO (population, intervention, comparison, outcome) framework and the study design [39]. After identifying relevant studies through the systematic database search, reference list screening was conducted by examining the reference lists of included studies [40].

For a detailed overview of the inclusion and exclusion criteria applied, see Textbox 1. The *population* included individuals identified as being unemployed, long-term unemployed, or job-seeking between the ages of 18 and 67 years. This age range was chosen since it indicates the population available for the labor market and covers the international distribution of retirement ages. Populations showing heightened levels of psychological distress were included. *Interventions* that were at least partly derived or modified from a psychological theory, a manual, or a therapeutic school (eg, cognitive behavioral therapy [CBT]) were included. Only studies with some type of control group were included. The following o*utcomes* were included: (1) subsyndromal psychological outcomes indicating well-being and stress, as well as syndrome-oriented outcomes such as measures of depression and anxiety, and (2) re-employment measured by re-employment rates or self-report.

**PICO (population, intervention, comparison, outcome) criteria applied for the inclusion of studies.**

**Inclusion criteria**

- Population: Individuals must meet at least one of the following conditions: (1) or (2) or (3), and (4)

1. Classified as long-term unemployed or jobless/job-seeking
2. Receiving social benefits
3. Experiencing psychological distress and recruited via social service institutions
4. Available for the labor market (age 18-67 years)

- Intervention: Interventions must include (1) or (2); Form of presentation: face-to-face or web-based/online; Intensity: any

1. Derived or modified from a psychological theory or manual
2. Interventions including individual placement and support but also incorporating psychological/psychotherapeutic or group therapy elements

- Comparison: Any kind of control group (eg, waitlist and treatment as usual)
- Outcome: Psychological outcomes (eg, well-being, distress, depression, and anxiety) and re-employment or employment status
- Study design: All kinds of pre-post interventions
- Language: English or German

**Exclusion criteria**

- Population: Individuals who are employed but have a low income, are sick-listed due to mental or physical disorders, are experiencing severe physical illness (eg, cancer) and have lost their job due to their health condition, are experiencing severe mental illness (eg, psychosis and acute addiction), are not primarily recruited from job agencies but from clinics or other institutions, and are younger than 18 years
- Intervention: Solely physical activity (eg, smoking prevention) or solely vocational training; Interventions exclusively targeting health behavior change (smoking, drinking, or exercising)
- Comparison: No control group
- Outcome: Publications without results; Publications reporting no psychological measures at all
- Study design: Case studies and qualitative data only
- Language: Not English or German

### Summarizing and Analyzing the Data

All references found were imported into an EndNote database [35]. Duplicates were deleted using the EndNote database tool in the first step. Further duplicates were deleted manually by the first author (SHA). All articles were selected using a multistage screening process supervised by a senior researcher (RE). All articles were first screened independently by title and abstract by 2 reviewers (SHA and RE) and categorized as “eligible” or “ineligible.” To assess interrater reliability, Cohen kappa was calculated. In the next step, potential studies for inclusion were assessed for eligibility on the basis of the full-text analyses (SHA and MM). In unclear cases or cases of diverging opinions, a senior researcher (FJ) was consulted.

If reported or applicable, data on intervention content and characteristics, study sample, and study results were extracted for each article (SHA and SS). In case of unclear information or lack of information, the authors were contacted. To evaluate the validity and quality of the included studies, risk of bias was assessed using the Revised Cochrane Risk-of-Bias Tool for Randomized Trials (RoB 2) [41] and the Risk of Bias in Nonrandomized Studies - of Interventions (ROBINS-I) tool [42]. In each case, the assessments were carried out in parallel by 2 independent researchers (SHA and MM).

In the case of studies classified as randomized controlled, content was rated as having *low risk of bias*, *some concerns*, or *high risk of bias* in the following domains: (1) randomization process, (2) deviations from intended interventions, (3) missing outcome data, (4) measurement of the outcome, and (5) selection of the reported result. Regarding the domain *risk of bias due to deviations from the intended interventions*, the subdomain *effect of adherence to intervention* was chosen to be examined as it presents a better fit to the overall question in the sense of a supply decision for an individual. For included randomized controlled trials, the overall risk-of-bias judgement was assessed as follows. A study was judged to be at low overall risk of bias if all 5 domains were judged as having low risk of bias. The overall judgment was “some concerns” if the study was rated to have some concerns in at least one of the domains. High risk of bias was applied if the study was rated to be at high risk of bias in at least one domain or if it was rated to have some concerns for multiple domains, causing a serious deterioration in the quality of the study.

A similar procedure was followed for nonrandomized controlled studies. In accordance with the guidelines by Sterne et al [42], studies were classified in the following bias domains: (1) selection of participants, (2) classification of interventions, (3) deviations from intended interventions, (4) missing data, (5) measurement of outcomes, and (6) selection of the reported result. Each domain was rated, resulting in overall judgements of low, moderate, serious, or critical risk of bias or no information available. A study was judged to be at an overall low risk of bias if all domains were at low risk. Moderate risk of bias was applied when the study was judged to be at low or moderate risk of bias for all domains. As blinding of both patients and treatment providers is difficult to achieve and maintain for nonpharmacological interventions [43], a higher risk of bias in this domain should be taken into consideration.

Overall serious risk was applied if a serious concern in at least one domain but no critical risk of bias for any domain was shown. No information at the overall level was applied in case of an unclear indication of serious or critical risk and a lack of information in one or more key domains of bias. The outcome variables to be investigated were defined in advance in collegial agreement and, if applicable, analyzed separately or grouped together. To visualize risk of bias, the robvis tool was used [36]. Differences were discussed between the researchers in the event of disagreement between raters. The senior researcher (RE) was consulted if the disagreement could not be resolved. Moreover, authors of the included studies were contacted in case clarifications were needed.

## Results

### Included Studies

**Figure 1:**
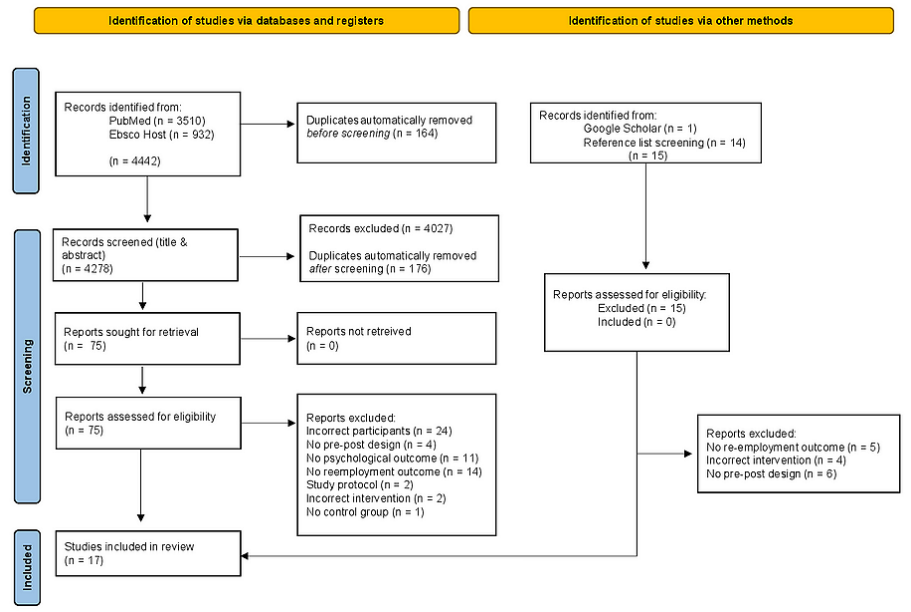
PRISMA (Preferred Reporting Items for Systematic Reviews and Meta-Analyses) flowchart.

The systematic electronic database search yielded 4442 articles. In addition, 15 articles were found via the manual search process (reference list screening). In 2 steps, 340 duplicates were removed, leaving 4278 articles to be screened. Of these articles, 4203 were excluded during the screening process. The interrater reliability was found to be good, with a Cohen κ of 0.770. The full texts of 74 articles were assessed for eligibility by the 2 independent reviewers, and furthermore, 58 articles were excluded, resulting in the final inclusion of 17 articles. The PRISMA flowchart is presented in Figure 1.

### Interventions to Promote Mental Health and Enhance Re-Employment Among Unemployed Individuals

To provide an answer to the first research question, “What types of interventions have been implemented to promote mental health and facilitate re-employment among unemployed individuals, and what are their characteristics and outcomes?”, we identified a diverse set of interventions across the included studies. The following section provides an overview of the included studies, summarizing their key characteristics, intervention types, and outcomes. Detailed overviews of the content, characteristics, and results of the studies are presented in Tables 1 and 2, and Multimedia Appendices 3 and 4. All included studies were published between 1989 and 2018. The size of the intervention groups ranged from 19 [44] to 1249 [45]. Studies were conducted in the United States [45-48], the Netherlands [49,50], Germany [51,52], Australia [44,53,54], Finland [55,56], the United Kingdom [57], and Ireland and the United Kingdom [58-60].

The target population was partly the general unemployed population [44,46,50,52,55-57,63] and partly unemployed people affected by multiple burdens, such as mentally distressed individuals [45,47-49,59], or otherwise disadvantaged individuals showing barriers to being placed in the labor market [51,53,54]. Moreover, the mean length of unemployment within the intervention groups ranged between 1 week [45] and 5 years [60].

The included studies were either randomized controlled studies [44-48,51,53,55-57,59] or nonrandomized studies that used a quasiexperimental design [49,50,52,54,58,60,63]. The studies also differed in terms of their recruitment methods. The most common and frequently used recruitment path was via the employees of employment agencies or respective institutions [49,50,52,54,58,60,63], followed by recruitment via the associated research team [45,46,48,49,55,56]. Proudfoot et al [57] used a different approach by placing an advertisement. A combination of methods (eg, advertisement and via an agency) was used by Hulshof [50] and Rothländer et al [52]. Several studies [45,48,55,56,58] used the principles of the JOBS program by Caplan et al [46], with either some country-specific [55,56,58] or content-specific adaptations [45,48]. The JOBS program is a structured, cognitive-behavioral intervention designed to improve job-seeking behavior and mental well-being among unemployed individuals. The program is grounded in the theory that unemployment is not only a financial issue but also a psychological one, where an individual’s sense of self-efficacy, motivation, and coping skills are negatively impacted. The JOBS program aims to enhance participants’ job-search self-efficacy, increase their motivation to seek work, and improve their mental health by addressing setbacks and building resilience. Moreover, 7 of the included trials used CBT-based interventions [44,47,52-54,57,59]. The remaining studies used either individualized approaches, such as interdisciplinary counseling [49], or individualized support by psychologists and physicians [51]. Hulshof [50] used means of positive psychology fostering psychological capital.

**Table 1:** Summary of the included interventions.

Study author (year), country	Intervention name and theoretical framework	Intervention type	Intervention format (length/setting)	Intervention focus and content
Barry et al [58] (2006), Ireland	Winning New Jobs Intervention; Based on the theory of JOBS [46,61]. Manual content adapted for the Irish population.	Face-to-face	5 half-day training workshops (20 hours)/groups of 12-22	Job-related skills and mental health promotion; Skilled trainers, who have been trained specifically to deliver the JOBS program, active learning, self-efficacy, sense of control and job-related self-efficacy, and inoculation against setbacks to deal with setbacks and obstacles.
Caplan et al [46] (1989), United States	JOBS I intervention; Intervention affects motivation and skills, which in turn can affect job-seeking behavior. Motivation is enhanced by prior success and undermined by setbacks.	Face-to-face	8 sessions of 3 hours each distributed over 2 weeks, 4 times per week (24 hours)/groups of 16-20	Improving job-related skills; Application of problem-solving and decision-making processes, inoculation against setbacks, receiving social support and positive regard from the trainers, and learning and practicing job-seeking skills.
Carlier et al [49] (2018), Netherlands	Vicious cycle of mental illness and unemployment; Unemployed individuals with mental health problems require both job-related and well-being–related interventions to be re-employed.	Face-to-face	Ongoing; maximum 2 years/individualized	Individual placement and support; Interdisciplinary teams counsel unemployed individuals toward employment. Addressing barriers to entering paid employment (eg, psychological problems and debt); providing psychological resources through coaching and cognitive counseling and employment activities tailored to the individual needs of the unemployed person.
Della-Posta et al [44] (2006), Australia	Effectiveness of cognitive behavioral therapy (CBT) to improve mental well-being; Improved well-being, in turn, supports successful re-employment.	Face-to-face	4 hours per week for 4 weeks; first 2 weeks: standard job search assistance; second 2 weeks: CBT (16 hours)/groups of approximately 5	Psychotherapy; Stress and relaxation, planning pleasant activities, communication, and positive and negative thinking; importance of clear communication and differences between passive, aggressive, and assertive styles of communication; simple breathing techniques and guided imagery to more effectively manage stress; incorporation of pleasant activities into their daily routine; and distinguishing between positive and negative thinking.
Harris et al [53] (2002), Australia	Effectiveness of CBT to improve mental well-being; The mental health of individuals who are particularly disadvantaged is more likely to be negatively affected by long-term unemployment; hence, an intervention supporting mental health is highly relevant to this sample.	Face-to-face	11 hours/groups of 5-16	Psychotherapy; Based on the intervention by Rose [54]; Cognitive restructuring, problem solving, and behavioral strategies including relaxation skills.
Herbig et al [51] (2012), Germany	AmigA-M; Highly individualized integration of job placement measures with health-related measures should lead to the improvement of mental health and re-employment.	Face-to-face	Over 6 months: average of 8 contacts with their respective case manager (duration of 44 min), 4 contacts with a physician (duration of 36 min), and 3 contacts with a psychotherapist (duration of 54 min)/individualized	Improving mental health and re-employment; Employment-oriented case management with a health-related focus, individualized support by psychologists and physicians by providing diagnostics, advisory support for the client, and decision-making aids for the case manager.
Himle et al [47] (2014), United States	Effectiveness of CBT for the treatment of individuals with social anxiety disorder (SAD) plus adding work-related content to CBT training to target mental health and unemployment.	Face-to-face	Eight 2-hour sessions held twice weekly over the course of 4 weeks (16 hours)/groups	Psychotherapy and job-related skills; Manualized group CBT for SAD by Heimberg and Becker [62]; elements of psychoeducation, cognitive restructuring, and exposure exercises; limited social skills training related to the work environment and standard vocational services.
Hulshof et al [63] (2020), Netherlands	Based on several theories: (1) conservation of the resources model (COR; people strive to protect resources, and well-being declines when people are threatened by the loss of valued resources); (2) job demands-resources model (JD-R [64]); (3) psychological capital (PsyCap) [65] stating that an individual’s positive psychological state of development is characterized by 4 elements: self-efficacy, optimism, hope, and resiliency; (4) experiential learning theory [66] and goal-setting theory [67,68]. Experiential learning theory emphasizes the importance of past experiences in learning and behavioral change. The 4 stages relevant for learning are all incorporated in the intervention.	Face-to-face	Three days (day 1: 5.5 hours; day 2: 5.5 hours; day 3: 3 hours) (14 hours)/groups of maximum 12 participants per group	Psychotherapy and job-related skills; Re-employment crafting, providing theory and practicing all aspects of PsyCap (eg, optimism by the positive visualization technique), theory and practicing with re-employment crafting (eg, setting SMART goals), and small homework assignments (eg, gratitude exercise and acts of kindness).
Maguire et al [59] (2014), United Kingdom	Effectiveness of CBT for mental health, which should, in turn, support employability plus adding work-related content to CBT training to target mental health and provide skills to obtain employment.	Face-to-face	10 days over 5 weeks	Psychotherapy and job-related skills; CBT-based exercises as well as job-seeking skills; key themes: CBT, self-determination theory, and career guidance.
Proudfoot et al [57] (1997), United Kingdom	CBT can support a healthy attributional style, which is affected by losing employment and failing to find re-employment.	Face-to-face	7 sessions of 3 hours, 1 per week over a 7-week period (21 hours)/groups of 10-15	Psychotherapy; Sessions: introduction to a cognitive model, automatic thoughts, goal-setting, time management, task breakdown, thought recording and common thinking errors, planning, techniques to change unhelpful thinking, gaining access to deeper beliefs, dimensions of attributional thinking, specific applications to personal and work situations, integration of strategies, action planning, and relapse prevention; assignment between sessions.
Reynolds et al [60] (2010), Ireland/United Kingdom	Theoretical framework of the JOBS program by Caplan et al [46]; The mechanisms include job search self-efficacy, sense of mastery, and inoculation against setbacks.	Face-to-face	5 sessions of 4 hours each distributed over 1-2 weeks (20 hours)/groups	Job-related skills; Based on Caplan et al [46] and Vinokur et al [45]: active teaching and learning methods, skilled trainers who have been trained specifically to deliver the JOBS program, and supportive learning environments; self-efficacy: training processes that enhance general confidence, sense of control and job-related self-efficacy for participants, and inoculation against setbacks.
Rose [54] (2001), Australia	Effectiveness of CBT to improve mental well-being.	Face-to-face	8 sessions over 2 days with 5.5 hours each (11 hours)/groups	Psychotherapy; “Strategies for re-employment” program; 3 core components: CBT skills, structured problem-solving, and relaxation skills.
Rothländer et al [52] (2012), Germany	AktivA; Effectiveness of CBT to improve mental well-being; Integration of the health action process approach [69] to consider the phases of health-related behavior (motivation, volition, and action).	Face-to-face	4 course days distributed over 2-4 weeks (24 hours)/groups	Psychotherapy; Elements of CBT: planning of activities, constructive thinking, social competencies and social support, and systematic problem-solving.
Vinokur et al [45] (1995), United States	JOBS II intervention (based on Caplan et al [46]).	Face-to-face	5 sessions of 4 hours each distributed over 1 week (20 hours)/groups of 12-22	Improving job-related skills; Application of problem-solving and decision-making group processes, inoculation against setbacks, provision of social support and positive regard from the trainers, and learning and practicing job search skills; 2 trainers (male/female) per group.
Vinokur et al [48] (2000), United States	Referring to Caplan et al [46] and Vinokur et al [45] - JOBS and JOBS II intervention.	Face-to-face	5 sessions of 4 hours each distributed over 1 week (20 hours)/groups of 12-22	Job-related skills; Based on Caplan et al [46] and Vinokur et al [45]: application of problem-solving and decision-making group processes, inoculation against setbacks, provision of social support and positive regard from the trainers, and learning and practicing job search skills; 2 trainers (male/female) per group.
Vuori et al [56] (2002), Finland	Työhön Job Search Program; Transferring JOBS II [46] to the European/Finnish context with minor procedural changes.	Face-to-face	5 sessions of 4 hours each distributed over 1 week (morning sessions; 20 hours)/groups of 6-17	Job-related skills; The training is designed to increase participants’ job-search self-efficacy and motivation as well as to enhance the following job-search skills: recognizing and communicating one’s marketable skills, identifying and using one’s social network to find job openings, contacting promising employers, drawing up a job application and resume, and preparing for successful job interviews.
Vuori et al [55] (2005), Finland	2-year follow-up of the study by Vuori et al [56].	Face-to-face	5 sessions of 4 hours each distributed over 1 week (morning sessions; 20 hours)/groups of 6-17	Job-related skills; The training is designed to increase participants’ job-search self-efficacy and motivation as well as to enhance the following job-search skills: recognizing and communicating one’s marketable skills, identifying and using one’s social network to find job openings, contacting promising employers, drawing up a job application and resume, and preparing for successful job interviews.

Outcome measures included the assessment of depression, distress, and well-being. A detailed overview of the instruments used is presented in Multimedia Appendix 5.

The way in which re-employment was assessed varied from using self-report measures for hours worked per week [45-48,51,54-56,59] to using objectively assessed data, for instance, when data on re-employment was provided by a job agency [44,49-51,63]. In some studies, further information was not provided on the source of the data on re-employment and additional information could not be obtained from the authors [52,53,57,58]. Parts of the interventions mostly focused on re-employment, for instance, by using methods to improve specific job search skills and inoculation against setbacks [45,46,48,55,56,60] or via methods from the field of individual placement and support [49]. On the other hand, some interventions were more focused on the improvement of mental health outcomes, for example, by using target group–adapted CBT-specific content such as planning euthymic activities, cognitive reconstruction, problem solving, social skills, and relaxation methods [44,52-54,57]. Some studies focused on both re-employment and mental health improvement [47,50,51,59,63].

The interventions varied in their theoretical foundations, delivery formats, and target outcomes, yet several overarching patterns emerged. CBT-based interventions (eg, [44,53,57,59]) primarily focus on improving psychological well-being, often through cognitive restructuring, stress management, and problem-solving strategies. These interventions have demonstrated positive effects on mental health, but their direct impact on re-employment remains inconsistent. In contrast, job-search training programs (eg, [45,46,60,70]) emphasize enhancing job-seeking self-efficacy and resilience against setbacks. These interventions tend to show stronger effects on re-employment rates. Additionally, integrative approaches, such as individualized case management (eg, [51]) or interventions combining psychotherapy with job-related skills development (eg, [47,63]), seek to address both psychological and employment-related barriers. While such integrative models may offer a more comprehensive support system, their effectiveness in improving employment outcomes remains variable.

**Table 2:** Summary of the included studies with an overview of the study methodologies and key results.

Study author (year), country	Control condition	Study design	Recruitment path	Results on mental health	Results on re-employment
Barry et al [58] (2006), Ireland	Treatment as usual (TAU)	Quasiexperimental design	Via employment agencies	No significant difference in depressive symptoms (for both measurement points).	At the 4-month follow-up: significant increase in re-employment rates for the intervention group (IG); At the 12-month follow-up: the program effect on re-employment was maintained (*P*<.001).
Caplan et al [46] (1989), United States	Active control group (CG) Received a brief booklet with job-seeking tips	Randomized field experiment	Directly via researchers within job centers	Whole sample: no significant findings; Comparison of re-employed and unemployed: re-employed had lower values for depression and anger.	Whole sample: status of re-employment: significant at T2 and T3 (greater percentage of re-employment in the IG); Re-employed sample: higher percentage in the IG for the main occupation.
Carlier et al [49] (2018), Netherlands	Passive control (TAU) Participation in usual re-employment programs (with various foci) Maximum duration of 1 year	Quasiexperimental design	Directly via a researcher (contact information provided by an employment center)	Physical health: no significant difference; Mental health: no significant difference; Anxiety and depressive symptoms: no significant difference.	No significant difference.
Della-Posta et al [44] (2006), Australia	Active CG Standard job search assistance group (16 hours; 4 hours for 4 weeks)	Quasiexperimental design	Via a vocational rehabilitation center	Depressive symptoms: no significant difference.	The IG shows re-employment within a shorter amount of time (at T1 and T2); no significant difference at the T3 follow-up.
Harris et al [53] (2002), Australia	Active CG First aid course	Controlled trial design with pre-post measures	Indirectly via employment counselors	Mental health: no significant difference.	No significant difference.
Herbig et al [51] (2012), Germany	Waitlist CG	Randomized controlled trial	Via employment agencies	Significant changes in depressive syndromes in the IG. More participants in the IG than in the CG showed improvements in major depressive syndrome.	No significant intervention effect on re-employment.
Himle et al [47] (2014), United States	TAU as vocational service as usual (VSAU); Contents amongst others: career assessment, résumé construction, job interviewing skills, job placement assistance	Randomized field experiment	Via a vocational rehabilitation center	Significant differences on nearly all scales in predicted direction, with the IG reporting better mental health and reduced social anxiety symptoms.	No significant difference.
Hulshof et al [63] (2020), Netherlands	Waitlist CG	Pre-posttest design	Invitation by the first author and via emails and letters sent by the unemployment agency	Significant time × group effect of the intervention between groups for negative affect (*F*=5.39; *P*<.001).	No significant intervention effect on re-employment.
Maguire et al [59] (2014), United Kingdom	Waitlist CG	Randomized, waitlist controlled design	Indirectly via referral from job centers, general practitioners, employment counselors, and further sources	Depressive symptoms: T2: significant difference with lower values in the IG; T3: significant difference with lower values in the IG.	T3: 20 individuals found employment; not statistically tested.
Proudfoot et al [57] (1997), United Kingdom	Active CG (social support program of the same duration); Sessions: (1) concept of social support, health, and unemployment; (2) life satisfaction graphs, peaks and troughs, and importance of people; (3) role-mapping, satisfying relationships, and personal support networks; (4) social awareness, how to create a positive impression, and rules of relationships; (5) listening and conversation skills; (6) people as resources; (7) goal-setting and course summary plus assignment between sessions	Controlled, experimental, 2-group design	Advertising study	Mental health: significant differences at T2 and T3.	Significant differences at T3: IG more likely to find a job than CG (T2 not relevant as directly at the end of training).
Reynolds et al [60] (2010), Ireland/United Kingdom	TAU in training and employment agencies	Quasiexperimental design	IG: selected by training and employment agencies, mostly “difficult to place;” CG: recruited by researchers within the same agencies	Depressive symptoms: no significant difference.	Significant difference: higher percentage of re-employment in the IG.
Rose [54] (2001), Australia	Active CG First aid course	Randomized controlled trial experimental design	Indirectly via employment consultants	Mental health: no significant difference	No significant difference in the proportion of recruits achieving a positive employment outcome in the IG and CG; employment-related status was recoded as “work and study” or “no work and study.”
Rothländer et al [52] (2012), Germany	TAU: participation in employment measures that do not include AktivA elements	Quasiexperimental design	IG 1: intervention integrated in employment measures of the employment agency; IG 2: intervention freely advertised in newspapers and posters	Health/well-being - psychological: T1-T2: IG 1: significant difference with lower levels of psychological complaints in the IG; IG 2: significant difference with lower levels of psychological complaints in the IG; T1-T3: IG 1: no significant difference (no time × group interaction); IG 2: significant time × group interaction: benefits of training persist mid-term.	No significant difference in the frequency of being unemployed.
Vinokur et al [45] (1995), United States	Active CG Received a brief booklet with job-seeking tips	Randomized field experiment	Directly via researchers within job centers	Depressive symptoms: significant interaction at T2 and T3; No significant difference for low-risk participants compared to the CG; Lower levels of depressive symptoms in the high-risk IG compared to the high-risk CG.	Significant at T2, interaction at T3; T2: re-employment higher in the IG (independent of risk status); T3: interaction of group and risk status: no significant difference between the CG and low-risk IG, but higher re-employment for the high-risk IG compared to the CG.
Vinokur et al [48] (2000), United States	Active CG Received a brief booklet with job-seeking tips	Randomized field experiment	Directly via researchers within job centers	Long-term effects of the intervention; significant beneficial effects on depressive symptoms.	Intervention had significant beneficial effects on being employed (>20 hours per week).
Vuori et al [56] (2002), Finland	Active CG CG received a literature package, which corresponded to the basic themes in job-search training and included four guides: (1) a guide to the services of the employment agency, (2) a guide to manage one’s life situation while unemployed, (3) an ABC guide for the job seeker, and (4) a handout of job-seeking advice	Randomized field experiment	Directly via researchers within job centers and outside	No significant effect for depressive symptoms.	Direct effect of the intervention at T3: no significant effect on re-employment rate, wage rate, job, and satisfaction; significant effect on stable re-employment: higher rate of re-employment in stable jobs in the IG.
Vuori et al [55] (2005), Finland	Active CG CG received a literature package, which corresponded to the basic themes in job-search training and included four guides: (1) a guide to the services of the employment agency, (2) a guide to manage one’s life situation while unemployed, (3) an ABC guide for the job seeker, and (4) a handout of job-seeking advice	Randomized field experiment	Directly via researchers within job centers and outside	Depressive symptoms: intervention status significantly and negatively predicted depressive symptoms.	No significant difference.

### Effects on Mental Health and Re-Employment

To address the second research question, “How effective are these interventions in enhancing mental health outcomes and improving re-employment rates?”, we examined the effects of each intervention on both mental health and re-employment outcomes. Relevant mental health outcome criteria were defined in advance by the authors.

Significant improvements in mental health outcomes in the intervention groups after receiving the program were shown in 9 of the 17 studies [45,47,48,51,52,55-57,59]. It should be noted that the studies by Vinokur et al [48] and Vuori et al [71] are follow-up measurements of the respective studies and thus show the long-term effects of the interventions. The study by Hulshof et al [50] shows the significant effects on mental health in the intervention group; however, this was due to an increase in negative affect in the control group.

Regarding re-employment, improvements in the intervention group in terms of an increased rate of re-employment after receiving the intervention were shown in 7 of the 17 studies [44-46,48,57,58,60]. In 9 of the 17 studies, no significant difference could be shown [47,49-56,63]. Maguire et al [59] reported that 20 individuals found employment; however, this finding was not statistically tested, and thus, it remains unclear if the intervention had a significant effect. Vuori et al [56] reported no significant effect on re-employment; however, significant effects on higher rates of re-employment in stable jobs in the intervention group were shown.

Moreover, several studies have reported challenges in the implementation of programs related to cooperation with participating organizations and institutions. On the one hand, this concerns aspects of the research design, since in many cases, randomization was not desired or feasible on the part of the agencies [53,58,60]. On the other hand, a lack of cooperation on the part of the agencies or the authorities was also reported, which led to deficient implementation [54]. Moreover, in some cases, further problems in the recruitment process due to the participants or their illness-related characteristics were described [57].

### Risk of Bias in the Included Studies

A detailed overview of all the ratings and judgements can be found in Multimedia Appendix 6.

#### Randomized Controlled Trials

Regarding randomized controlled trials, the overall quality was rated as “low risk” in 1 article [57], indicating a low risk of bias in all domains. Moreover, 9 articles [44-48,51,53,55,56] received an overall rating of “some concerns” (some concerns in at least one domain; no domain judged to have a high risk of bias). Furthermore, 1 article [59] was judged to be at high risk of bias (defined as having a high risk of bias in at least one domain or as having some concerns for multiple domains). Moderate or weak ratings mainly resulted from risk of bias arising from the randomization process or due to no information in relevant domains, especially regarding the selection of reported results.

#### Nonrandomized Trials

One article [63] was judged to be at an overall moderate risk of bias (low or moderate risk of bias for all domains). Three articles [52,54,60] were found to have a serious risk of bias (serious risk of bias in at least one domain, but not at critical risk of bias in any domain). Lastly, 1 article [49] was found to have a critical risk of bias (critical risk of bias in at least one domain). One article [58] had an overall judgement of “no information” on which to base a judgement about risk of bias. Critical and serious ratings mainly resulted from bias due to the selection of participants and missing data.

**Figure 2:**
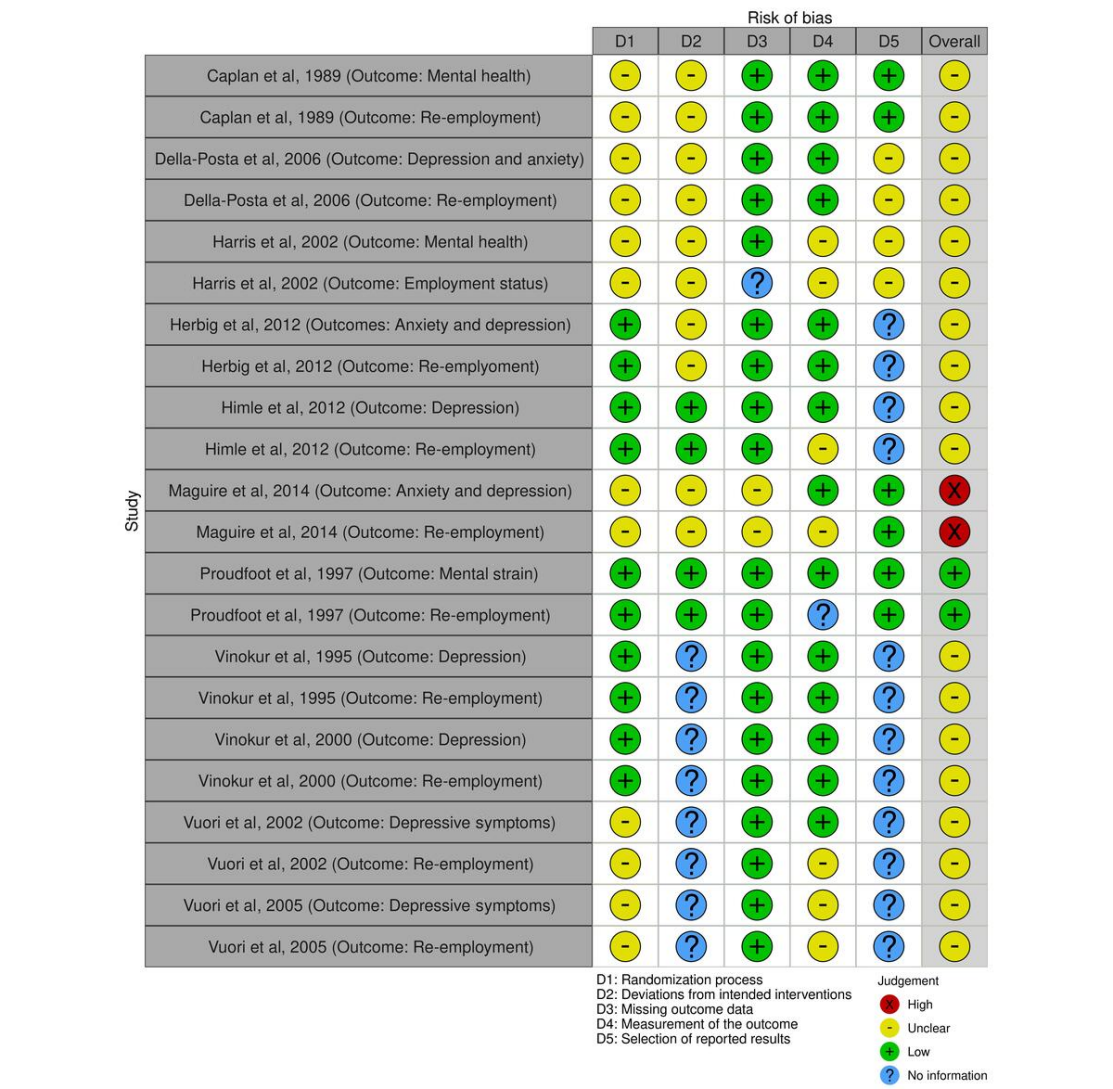
Visualized risk of bias assessment for the included randomized controlled trials [44-48,51,53,55-57,59], evaluating 5 domains: randomization process (D1), deviations from intended interventions (D2), missing outcome data (D3), measurement of the outcome (D4), and selection of reported results (D5). Each study has been assessed for mental health and re-employment outcomes.

**Figure 3:**
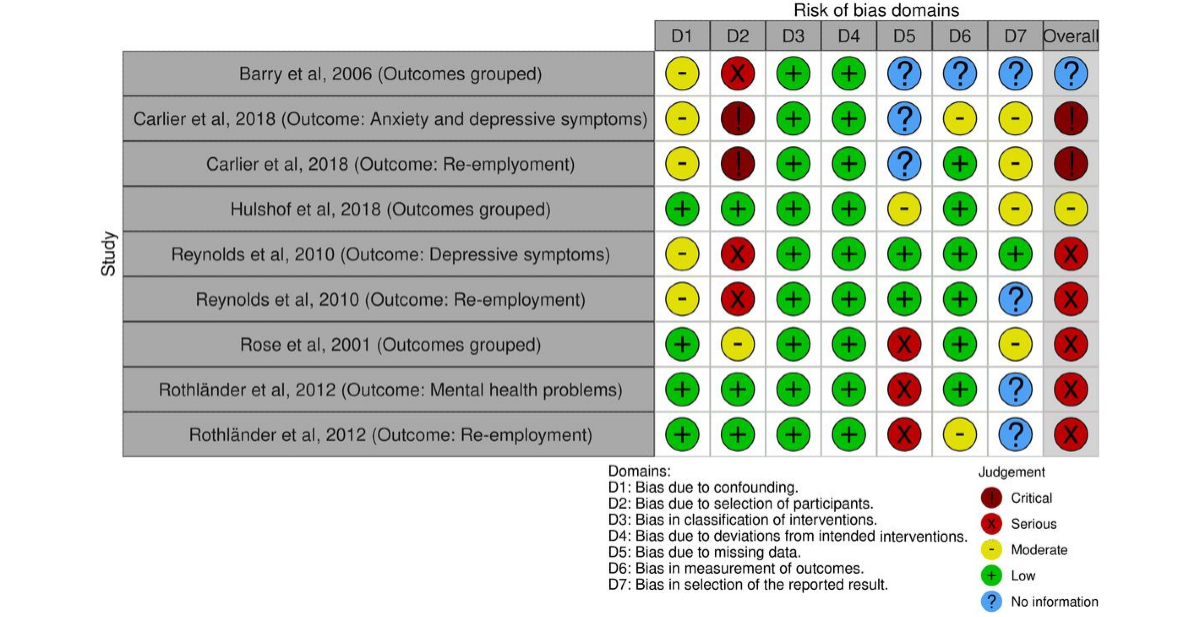
Visualized risk of bias assessment for the included nonrandomized controlled trials [49,52,54,58,60,61], evaluating the following domains: bias due to confounding (D1), bias due to selection of participants (D2), bias in classification of interventions (D3), bias due to deviations from intended interventions (D4), bias due to missing data (D5), bias in measurement of outcomes (D6), and bias in selection of the reported result (D7). Each study is assessed for mental health and re-employment outcomes.

**Figure 4:**
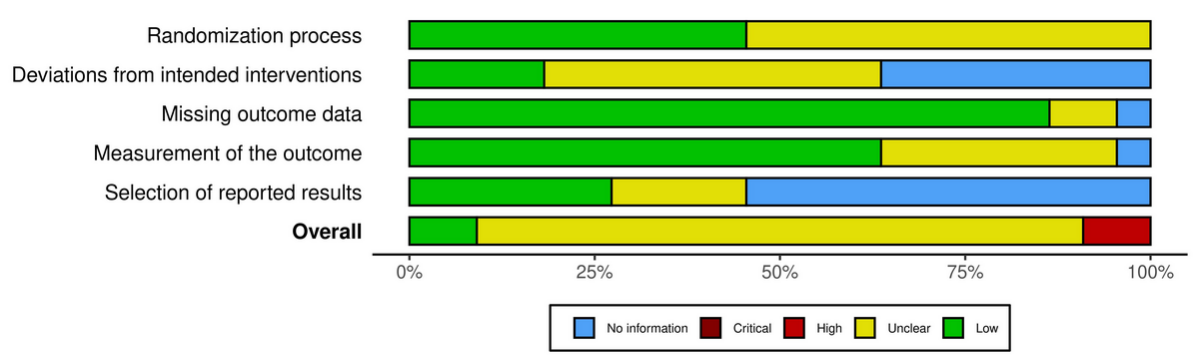
Visualized summary of risk of bias across all included randomized controlled trials, categorized by 5 domains: randomization process, deviations from intended interventions, missing outcome data, measurement of the outcome, and selection of reported results, as well as the overall risk of bias. The proportions of the risk categories are displayed as horizontal bars, providing an overview of the methodological quality of the included studies.

**Figure 5:**
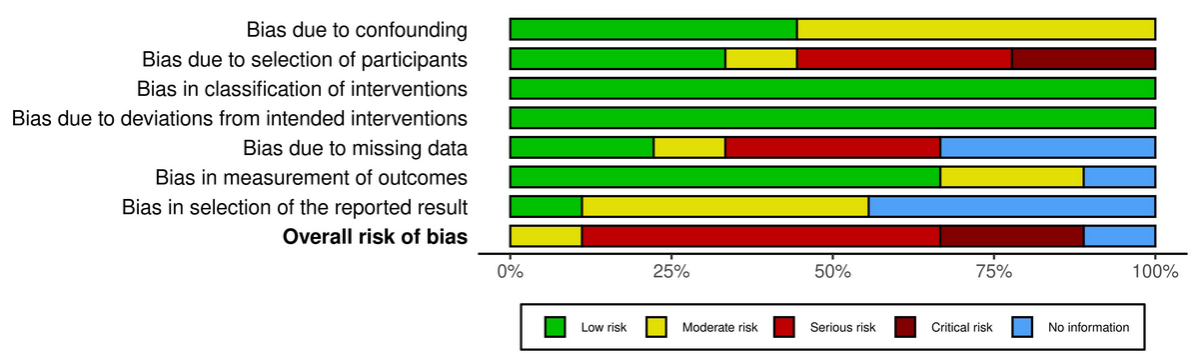
Visualized summary of risk of bias across all included nonrandomized controlled trials, categorized by the following bias domains: bias due to confounding, bias due to selection of participants, bias in classification of interventions, bias due to deviations from intended interventions, bias due to missing data, bias in measurement of outcomes, and bias in selection of the reported result, as well as the overall risk of bias. The proportions of the risk categories are displayed as horizontal bars, providing an overview of the methodological quality of the included studies.

Plots of the domain-level judgements for each individual result and each outcome are provided in Figures 2 and 3. Figures 4 and 5 display the overall percentage of judgements for each of the criteria [36].

## Discussion

### Evolving Integrated Intervention Approaches to Support Mental Health and Re-Employment

Unemployment remains a significant societal issue with profound psychosocial consequences that impact both individuals and communities. As individuals experiencing unemployment often face compounded barriers in finding work and accessing support, this population remains severely underserved. To the best of our knowledge, this review is the first to synthesize interventions aimed at improving both mental health outcomes and re-employment rates for individuals facing unemployment and psychological distress. Regarding research question 1 (*What types of interventions have been implemented to promote mental health and facilitate re-employment among unemployed individuals, and what are their characteristics and outcomes?*), we could identify content and methodologically heterogeneous approaches, which are mostly delivered in groups, and the access paths are typically provided through institutions (eg, job centers and employment agencies). It can also be seen that over the period from 1989 to 2018, interventions targeting re-employment and mental health among unemployed individuals have undergone several key evolutionary shifts. Early interventions, such as the JOBS program developed by Caplan et al [46], primarily focused on improving job-seeking skills and self-efficacy, and providing emotional support to individuals facing unemployment. These programs were structured around in-person, face-to-face workshops and sessions, often using methods like problem-solving, decision-making, and inoculation against setbacks. By the late 1990s and 2000s, CBT began to play a larger role in interventions. This shift recognized the importance of addressing mental health alongside job-related skills. More recent interventions (eg, [49,51]) have become more individualized, offering personalized coaching and support tailored to the specific needs of each participant. These programs also include more diverse formats, such as ongoing, long-term support rather than short-term interventions. Moreover, interventions, such as those by Hulshof [50] and Maguire et al [59], emphasize building psychological resources, such as resilience, hope, and optimism, alongside the development of job-related skills. These programs recognize that participants need to not only learn job-seeking strategies but also develop positive psychological attributes that can help them cope with the challenges of re-employment. These trends highlight the growing sophistication of employment interventions, from basic skills training to comprehensive, personalized programs that integrate mental health and psychological resilience.

Regarding research question 2 (*How effective are these interventions in enhancing mental health outcomes and improving re-employment rates?*), the results indicate that work-oriented interventions can have positive effects on mental health outcomes. This is consistent with the findings of Barry et al [58], who identified an enhanced sense of mastery as a possible mechanism of change for financial strain and depression symptoms. In turn, Della-Posta and Drummond [44] found that interventions that focus on mental health, for instance, via CBT-based interventions, can have a beneficial influence on re-employment. Most of the studies employed group settings where individuals shared their experiences with unemployment and the associated distress. Given the aim to provide a supportive environment for learning and developing coping strategies, group- and self-help–oriented approaches hold great potential for this target group. Additionally, this approach can help alleviate feelings of loneliness and social exclusion that often accompany unemployment [72], promoting social inclusion within a peer group [46,49-51,59]. Furthermore, research has shown associations between unemployment and increased mental health costs [73,74]. Therefore, it is important to consider macro-level factors associated with mental disorders, such as the considerable economic burden for respective countries [75]. Additionally, nonmonetary aspects, such as the number of disability-adjusted life years, should not be underestimated as they are among the leading causes of burden worldwide [76].

Based on the findings of this review, that is, the heterogeneity in intervention content among the included studies and their effects on respective outcomes, it is advisable to provide personalized and low-threshold care for individuals experiencing unemployment and psychological distress.

The following content and overarching themes can serve as a guideline for content options for integrative support measures. At the level of psychological and therapeutical support, this might be (1) promoting motivation for change, (2) prioritizing crisis interventions, (3) fostering the identification of dysfunctional thought patterns and learned helplessness and recognizing work-specific anxiety-inducing negative experiences (eg, with colleagues or superiors), (4) giving assistance in maintaining a daily schedule despite periods of unemployment, (5) fostering and reactivating social support using group settings, (6) strengthening psychosocial resources, (7) support in transitioning into the respective care system, and (8) maintaining contact beyond the time of re-employment, if possible. At the job-search level, the increased use of supported employment-oriented approaches [77] represents a possible innovative approach as it considers personal mental health limitations and close cooperation with employers and continues to provide further support beyond the time of re-employment. Moreover, including and reactivating the individual’s social network to leverage support in the search for new employment opportunities may serve as a possible approach. This review focuses on interventions that are accessible through job agencies or other publicly accessible pathways, with an emphasis on their preventive nature in breaking the vicious cycle of poor mental health and unemployment. Thus, the consideration of structural levels might also be needed. To enhance collaboration and effectiveness, it is important to maximize synergies with existing support systems and cooperatively integrate them. For instance, job centers can engage in exchange with other supportive stakeholders.

### Limitations

This review has some limitations. Although we used the RoB 2 tool according to the recommendations in the current Cochrane Handbook for Systematic Reviews of Interventions [41], we acknowledge that this tool has been reported to have low interrater reliability [78], and challenges in using the tool consistently may make it difficult to interpret the risk of bias ratings. A limitation of interpretability is the identified heterogeneity of the samples, especially in terms of the length of unemployment (see Multimedia Appendix 4) since longer periods of unemployment can lead to a decline in mental health, with some stabilization occurring at a low level in the second year of unemployment [6].

Thus, the comparability of intervention effects may be restricted due to heterogeneity in other characteristics, such as the composition of the population, differences between countries, and the content of the intervention. It is important to consider these factors as part of the overall analysis. It is also important to consider that this review synthesizes findings from the last 30 years. During this time, the diagnosis and treatment of mental disorders, the general perception of mental health, and economic circumstances, such as economic crises accompanied by short-term contracts and general job insecurity, may have influenced the overall economy and the well-being of the population [79]. It is also important to note that only the re-employment rate was used in the synthesis of the results presented here, and therefore, other criteria used in many studies, such as the quality of a new job, were not included.

### Future Perspectives

Future studies should include longer follow-up intervals to adequately assess the long-term effects of interventions on psychological outcomes and re-employment. Moreover, based on the described problems in the implementation of well-designed interventions, close interdisciplinary cooperation with different support structures and facilitators is needed [80]. It might be assumed that the external circumstances are more difficult and that the studies place an additional burden on the agencies; this should be increasingly addressed in future studies. In addition, future research should address the problems of recruitment and ways to increase participant adherence to interventions.

**Figure 6:**
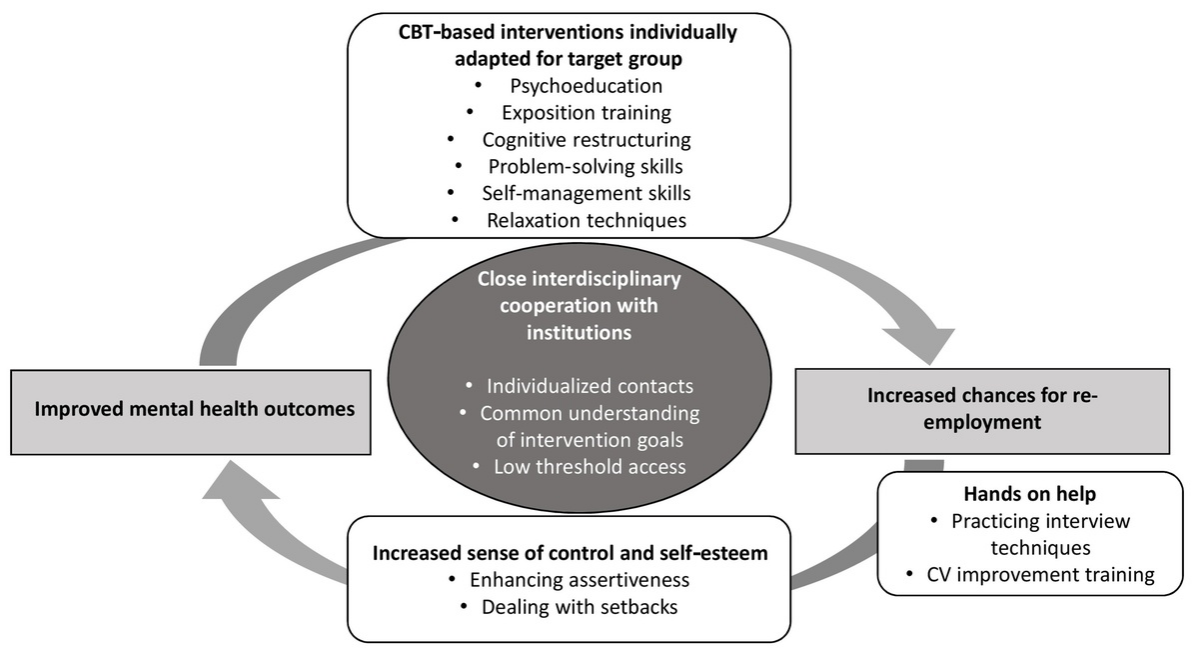
Synthesized results of evidence-based implications for research and practice. Dark grey: aspects to be considered in cooperation with executing and cooperating institutions; light grey: target outcomes; white: specific interventions or intervention components that emerge from the systematic review as contributing to improvements. CBT: cognitive behavioral therapy; CV: curriculum vitae.

For an overview of the implications for research and practice, see Figure 6.

The results of the studies, especially regarding re-employment, must be viewed in their sociocultural context and the associated opportunities to find labor. Future studies should also address the cost economics of interventions with novelty value and provide a trade-off between monetary costs and the added value of interventions. Finally, it is important to maintain the methodological quality of interventions, especially in the field of health services research, to ensure their scientific added value [81]. It can also be noted that improvements in aspects can positively transfer to one another. Thus, the respective aims are not strictly separable, and for instance, positive job search experiences may contribute to affective improvement without being explicitly targeted by psychotherapeutic interventions.

## Data Availability

All data generated or analyzed during this study are included in this published article and its supplementary information files.

## Appendix

Multimedia Appendix 1 PRISMA checklist.

Multimedia Appendix 2 Search terms.

Multimedia Appendix 3 Graphical overview of the results on mental health and re-employment in each included study.

Multimedia Appendix 4 Sample characteristics of the included studies.

Multimedia Appendix 5 Overview of the measurement instruments used.

Multimedia Appendix 6 Detailed overview of all ratings and judgements.

## Abbreviations

CBT: cognitive behavioral therapy
PRISMA: Preferred Reporting Items for Systematic Reviews and Meta-Analyses
RoB 2: Risk-of-Bias Tool for Randomized Trials
ROBINS-I: Risk of Bias in Nonrandomized Studies - of Interventions
