# Supplementary material for: Interventions to Foster Mental Health and Reintegration in Individuals Who Are Unemployed: Systematic Review
Source: JMIR Public Health Surveill. 2025 May 5;11:e65698. doi: 10.2196/65698 (PMC12089865; doi:10.2196/65698)
Supplement: Multimedia Appendix 2 [file publichealth_v11i1e65698_app2.docx]

**Multimedia Appendix 1.** Search terms.

Search Term created for PubMed:

(intervention* OR program* OR treatment* OR counsel* OR psychotherap* OR training OR web OR internet OR online) AND (unemploy* OR job-seek* OR well-fare OR job-loss) AND (mental health OR psych* OR well-being OR wellbeing OR self-effic* OR depression OR anxiety* OR stress OR distress) AND (RCT OR randomi* OR evaluation OR trial).

All other databases were searched using the following term:

(intervention OR treatment OR therap* OR counsel* OR program* OR course OR training OR web OR “online therapy” OR “internet-based or psychoeducation” AND (unemploy* OR jobless OR job loss OR job-seeking OR well-fare) AND (randomized controlled trials OR rtc OR randomized control trials OR trial OR evaluation) AND (mental health OR mental illness OR mental disorder OR psychiatric illness OR distress OR depress* OR anxiety OR wellbeing OR well-being OR self-efficacy OR re-employment).
