# Supplementary material for: Interventions to Foster Mental Health and Reintegration in Individuals Who Are Unemployed: Systematic Review
Source: JMIR Public Health Surveill. 2025 May 5;11:e65698. doi: 10.2196/65698 (PMC12089865; doi:10.2196/65698)
Supplement: Multimedia Appendix 3 [file publichealth_v11i1e65698_app3.docx]

**Multimedia Appendix 2.** Graphical overview of the results on mental health and re-employment in each included study.

| **First author**  **(year of publication),** | Intervention focus (Mental health / re-employment / both) | Mental Health | Re-employment |
| --- | --- | --- | --- |
| Barry (2006) | both | 🗶 | ✓ |
| Caplan (1989) | Re-employment | 🗶 | ✓ |
| Carlier (2018) | Re-employment | 🗶 | 🗶 |
| Della-Posta (2006) | Mental health | 🗶 | ✓ |
| Harris (2002) | Mental health | 🗶 | 🗶 |
| Herbig (2012) | both | ✓ | 🗶 |
| Himle (2014) | Mental health | ✓ | 🗶 |
| Hulshof (2020) | both | 🗶 | 🗶 |
| Maguire (2014) | Mental health | ✓ | * |
| Proudfoot (1997) | Mental health | ✓ | ✓ |
| Reynolds (2010) | Re-employment | 🗶 | ✓ |
| Rose (2001) | Mental health | 🗶 | 🗶 |
| Rothländer (2012) | Mental health | ✓ | 🗶 |
| Vinokur (1995) | Re-employment | ✓ | ✓ |
| Vinokur (2000) | Re-employment | ✓ | ✓ |
| Vuori (2002) | Re-employment | ✓ | 🗶 |
| Vuori (2005) | Re-employment | ✓ | 🗶 |

*Note*. 🗶: No significant result found in the intervention group with regard to the respective predefined outcome criteria; ✓: significant result found in the intervention group with regard to the respective predefined outcome criteria indicating a significant intervention effect; *results were statistically tested.

Reference List:

Barry M, Reynolds C, Sheridan A, Egenton R. Implementation of the JOBS programme in Ireland. Journal of Public Mental Health. 2006.

Caplan RD, Vinokur AD, Price RH, van Ryn M. Job seeking, reemployment, and mental health: a randomized field experiment in coping with job loss. J Appl Psychol. 1989 Oct;74(5):759-69. PMID: 2793774. doi: <https://10.1037/0021-9010.74.5.759>.

Carlier BE, Schuring M, Burdorf A. Influence of an Interdisciplinary Re-employment Programme Among Unemployed Persons with Mental Health Problems on Health, Social Participation and Paid Employment. J Occup Rehabil. 2018 Mar;28(1):147-57. PMID: 28397017. doi: <https://10.1007/s10926-017-9704-3>.

Della-Posta C, Drummond PD. Cognitive behavioural therapy increases re-employment of job seeking worker's compensation clients. J Occup Rehabil. 2006 Jun;16(2):223-30. PMID: 16705491. doi: <https://10.1007/s10926-006-9024-5>.

Harris E, Lum J, Rose V, Morrow M, Comino E, Harris M. Are CBT interventions effective with disadvantaged job-seekers who are long-term unemployed? Psychology, Health & Medicine. 2002;7(4):401-10. PMID: 2002-06931-004. doi: <https://10.1080/1354850021000015221>.

Herbig B, Glaser J, Angerer P. Old, sick, unemployed, without a chance? Results of a randomised controlled trial of the effects of a combined health and employment promotion program for the older long-term unemployed (AmigA-M). Bundesgesundheitsblatt Gesundheitsforschung Gesundheitsschutz. 2012 Aug;55(8):970-9. PMID: 22842891. doi: <https://10.1007/s00103-012-1514-3>.

Himle JA, Bybee D, Steinberger E, Laviolette WT, Weaver A, Vlnka S, et al. Work-related CBT versus vocational services as usual for unemployed persons with social anxiety disorder: A randomized controlled pilot trial. Behav Res Ther. 2014 Dec;63:169-76. PMID: 25461793. doi: <https://10.1016/j.brat.2014.10.005>.

Hulshof IL, Demerouti E, Le Blanc PM. Providing Services During Times of Change: Can Employees Maintain Their Levels of Empowerment, Work Engagement and Service Quality Through a Job Crafting Intervention? Front Psychol. 2020;11:87. PMID: 32047468. doi: <https://10.3389/fpsyg.2020.00087>.

Maguire N, Hughes VC, Bell L, Bogosian A, Hepworth C. An evaluation of the choices for well-being project. Psychol Health Med. 2014;19(3):303-15. PMID: 23822617. doi: <https://10.1080/13548506.2013.806813>.

Proudfoot J, Guest D, Carson J, Dunn G, Gray J. Effect of cognitive-behavioural training on job-finding among long-term unemployed people. Lancet. 1997 Jul 12;350(9071):96-100. PMID: 9228961. doi: <https://10.1016/s0140-6736(96)09097-6>.

Reynolds C, Barry MM, Gabhainn SN. Evaluating the impact of the winning new jobs programme on the re-employment and mental health of a mixed profile of unemployed people. International Journal of Mental Health Promotion. 2010;12(2):32-41. PMID: 2010-10753-006. doi: <https://10.1080/14623730.2010.9721812>.

Rose V. Improving the Health of People who are Unemployed through the Job Network. A study of a brief CBT intervention in South Western Sydney. 2001.

Rothländer K, Mühlpfordt S, Richter P. Evaluation des Gesundheitsförderungsprogramms 'Aktive Bewältigung von Arbeitslosigkeit(AktivA)' = Evaluation of the health promotion program 'Active Coping with Unemployment (AktivA)'. Zeitschrift für Gesundheitspsychologie. 2012;20(3):115-27. PMID: 2012-19868-002. doi: <https://10.1026/0943-8149/a000070>.

Vinokur AD, Price RH, Schul Y. Impact of the JOBS intervention on unemployed workers varying in risk for depression. Am J Community Psychol. 1995 Feb;23(1):39-74. PMID: 7572826. doi: <https://10.1007/bf02506922>.

Vinokur AD, Schul Y, Vuori J, Price RH. Two years after a job loss: long-term impact of the JOBS program on reemployment and mental health. J Occup Health Psychol. 2000 Jan;5(1):32-47. PMID: 10658883. doi: <https://10.1037//1076-8998.5.1.32>.

Vuori J, Silvonen J. The benefits of a preventive job search program on re‐employment and mental health at 2‐year follow‐up. Journal of Occupational and Organizational Psychology. 2005;78(1):43-52. doi: <https://doi.org/10.1348/096317904X23790>.

Vuori J, Silvonen J, Vinokur AD, Price RH. The Työhön Job Search Program in Finland: benefits for the unemployed with risk of depression or discouragement. J Occup Health Psychol. 2002 Jan;7(1):5-19. PMID: 11827233. doi: https://10.1037//1076-8998.7.1.5.
