# Supplementary material for: Interventions to Foster Mental Health and Reintegration in Individuals Who Are Unemployed: Systematic Review
Source: JMIR Public Health Surveill. 2025 May 5;11:e65698. doi: 10.2196/65698 (PMC12089865; doi:10.2196/65698)
Supplement: Multimedia Appendix 4 [file publichealth_v11i1e65698_app4.docx]

**Multimedia Appendix 3.** Sample characteristics of the included studies.

| **First author**  **(year of publication),**  **country implemented** | **Target population** | **Number of participants** | **Mean length of unemployment in sample** |
| --- | --- | --- | --- |
| Barry (2006), Ireland | ‘Opportunity and job ready’long- and short term unemployed  people and mental health service users | N = 437  IG: 245  CG:192 | M = 3.93 years |
| Caplan (1989), USA | Short-term unemployed individuals who receive unemployment compensation | IG:  T1: 606; T2: 412; T3: 414  CG:  T1: 322; T2: 281; T3: 214 | M = 13 weeks  SD = 9 weeks |
| Carlier (2018), Netherlands | Unemployed individuals with mental health problems | N = 158  IG: 51  CG: 107 | <1 year: 21.5% / 13.8%  1–5 years: 39.8% / 32.8%  >5 years or never worked: 38.7% / 53.8% |
| Della-Posta (2006), Australia | Individuals who receive redeployment  assistance and have been referred for a Job Search Programme as a part of their rehabilitation | N = 39  IG: 19  CG: 20 | Not stated |
| Harris (2002), Australia | Disadvantaged long-term unemployed individuals  Disadvantaged: Individuals have been assessed by the employment support agency using a common instrument  developed by the federal government as having identified ‘barriers’ to employment such as  limited education or training, long periods of unemployment or factors that placed them at risk for long-term unemployment | N = 195  IG: 123  CG: 72  Analysed sample:  N = 100  IG: 57  CG: 43 | M = 33 months (SD = 40.0)  Analysed sample:  M = 39 months (SD = 50.2) |
| Herbig (2012), Germany | long-term unemployed people with placement-relevant health and/or psychosocial restrictions older than 50 years | IG: 27  CG: 44 | IG: M = 1393,0 days (SD = 938,1)  CG: M = 1433,1 days (SD =712,1) |
| Himle (2014), USA | Unemployed, vocational service-seeking adults with social anxiety disorder | N = 58 (ITT)  IG: 29  CG: 29 | Not stated |
| Hulshof (2020), Netherlands | Unemployed individuals between 28 and 65 that are unemployed for at least four months | IG: 146  CG: 275 | M = 16.9 months (SD = 9.7 months) |
| Maguire (2014), UK | Unemployed individuals with mild to moderate mental health problem | N = 109 | 22.2% unemployed for less than 1 year;  25.0% unemployed between 1 and 3 years;  44.4%: unemployed for over 3 years;  Claiming benefits between 0 and 456 months |
| Proudfoot (1997), UK | Long-term unemployed individuals | N = 244  IG: 134  CG: 110 | IG: M = 25.8 months  CG: M = 23.1 months  (range <12 months to 12 years) |
| Reynolds (2010), Ireland /UK | Individuals that are unemployed, but ready to work;  focus in "difficult-to-place" individuals, including long-term unemployed | N = 352  IG: 162  CG: 190 | IG: M = 5.06 years (SD = 5.80)  CG: M = 3.09 years (SD = 4.05) |
| Rose (2001), Australia | Intensive assistance or “Flex 3” clients  registered with Job Network agencies in the South West and Central areas of Sydney  Intensive assistance: job seekers who are most disadvantaged in the labour market  to obtain and maintain employment | N = 195  IG: 123  CG: 72  Analysed sample:  N = 100  IG: 57  CG: 43 | 59.1% had been unemployed for more than 18 months and 57.7% had not engaged in any work  (including voluntary) in the last 3-months |
| Rothländer (2012), Germany | Unemployed individuals | N = 431  IG 1: 139  IG 2: 110  CG 1: 97  CG 2: 85 | IG 1/CG 1: mostly less than 1 year (41.5%/38.6%)  IG 2/CG 2: mostly less than 1 year (41.6%/32.8%) |
| Vinokur (1995), USA | Unemployed participants, particularly those in high risk of depressive symptoms  Job loss is recent (less than 13 weeks ago) | N = 1801  IG: 1249  CG: 552 | Between 1 and 13 weeks following job loss  (M = 4.11 weeks; SD = 3.8) |
| Vinokur (2000), USA | Short-term unemployed individuals  (when intervention was given; follow-up effects 2 years after intervention) | N = 1430  IG: 1249  CG: 552 | See Vinokur et al. (1995) |
| Vuori (2002), Finland | Unemployed individuals or individuals who recently received termination notice and are seeking employment | N = 1261  IG: 629  VG: 632 | M = 10.7 months (SD = 17.3)  Median = 17.3  28% > 12 months unemployed |
| Vuori (2005), Finland | Follow-up sample of Vuori et al. (2002) | N = 1144  IG: 629  CG: 632 | See Vuori et al. (2002) |

Reference List:

Barry M, Reynolds C, Sheridan A, Egenton R. Implementation of the JOBS programme in Ireland. Journal of Public Mental Health. 2006.

Caplan RD, Vinokur AD, Price RH, van Ryn M. Job seeking, reemployment, and mental health: a randomized field experiment in coping with job loss. J Appl Psychol. 1989 Oct;74(5):759-69. PMID: 2793774. doi: <https://10.1037/0021-9010.74.5.759>.

Carlier BE, Schuring M, Burdorf A. Influence of an Interdisciplinary Re-employment Programme Among Unemployed Persons with Mental Health Problems on Health, Social Participation and Paid Employment. J Occup Rehabil. 2018 Mar;28(1):147-57. PMID: 28397017. doi: <https://10.1007/s10926-017-9704-3>.

Della-Posta C, Drummond PD. Cognitive behavioural therapy increases re-employment of job seeking worker's compensation clients. J Occup Rehabil. 2006 Jun;16(2):223-30. PMID: 16705491. doi: <https://10.1007/s10926-006-9024-5>.

Harris E, Lum J, Rose V, Morrow M, Comino E, Harris M. Are CBT interventions effective with disadvantaged job-seekers who are long-term unemployed? Psychology, Health & Medicine. 2002;7(4):401-10. PMID: 2002-06931-004. doi: <https://10.1080/1354850021000015221>.

Herbig B, Glaser J, Angerer P. Old, sick, unemployed, without a chance? Results of a randomised controlled trial of the effects of a combined health and employment promotion program for the older long-term unemployed (AmigA-M). Bundesgesundheitsblatt Gesundheitsforschung Gesundheitsschutz. 2012 Aug;55(8):970-9. PMID: 22842891. doi: <https://10.1007/s00103-012-1514-3>.

Himle JA, Bybee D, Steinberger E, Laviolette WT, Weaver A, Vlnka S, et al. Work-related CBT versus vocational services as usual for unemployed persons with social anxiety disorder: A randomized controlled pilot trial. Behav Res Ther. 2014 Dec;63:169-76. PMID: 25461793. doi: <https://10.1016/j.brat.2014.10.005>.

Hulshof IL, Demerouti E, Le Blanc PM. Providing Services During Times of Change: Can Employees Maintain Their Levels of Empowerment, Work Engagement and Service Quality Through a Job Crafting Intervention? Front Psychol. 2020;11:87. PMID: 32047468. doi: <https://10.3389/fpsyg.2020.00087>.

Maguire N, Hughes VC, Bell L, Bogosian A, Hepworth C. An evaluation of the choices for well-being project. Psychol Health Med. 2014;19(3):303-15. PMID: 23822617. doi: <https://10.1080/13548506.2013.806813>.

Proudfoot J, Guest D, Carson J, Dunn G, Gray J. Effect of cognitive-behavioural training on job-finding among long-term unemployed people. Lancet. 1997 Jul 12;350(9071):96-100. PMID: 9228961. doi: <https://10.1016/s0140-6736(96)09097-6>.

Reynolds C, Barry MM, Gabhainn SN. Evaluating the impact of the winning new jobs programme on the re-employment and mental health of a mixed profile of unemployed people. International Journal of Mental Health Promotion. 2010;12(2):32-41. PMID: 2010-10753-006. doi: <https://10.1080/14623730.2010.9721812>.

Rose V. Improving the Health of People who are Unemployed through the Job Network. A study of a brief CBT intervention in South Western Sydney. 2001.

Rothländer K, Mühlpfordt S, Richter P. Evaluation des Gesundheitsförderungsprogramms 'Aktive Bewältigung von Arbeitslosigkeit(AktivA)' = Evaluation of the health promotion program 'Active Coping with Unemployment (AktivA)'. Zeitschrift für Gesundheitspsychologie. 2012;20(3):115-27. PMID: 2012-19868-002. doi: <https://10.1026/0943-8149/a000070>.

Vinokur AD, Price RH, Schul Y. Impact of the JOBS intervention on unemployed workers varying in risk for depression. Am J Community Psychol. 1995 Feb;23(1):39-74. PMID: 7572826. doi: <https://10.1007/bf02506922>.

Vinokur AD, Schul Y, Vuori J, Price RH. Two years after a job loss: long-term impact of the JOBS program on reemployment and mental health. J Occup Health Psychol. 2000 Jan;5(1):32-47. PMID: 10658883. doi: <https://10.1037//1076-8998.5.1.32>.

Vuori J, Silvonen J. The benefits of a preventive job search program on re‐employment and mental health at 2‐year follow‐up. Journal of Occupational and Organizational Psychology. 2005;78(1):43-52. doi: <https://doi.org/10.1348/096317904X23790>.

Vuori J, Silvonen J, Vinokur AD, Price RH. The Työhön Job Search Program in Finland: benefits for the unemployed with risk of depression or discouragement. J Occup Health Psychol. 2002 Jan;7(1):5-19. PMID: 11827233. doi: https://10.1037//1076-8998.7.1.5.
