# Supplementary material for: Interventions to Foster Mental Health and Reintegration in Individuals Who Are Unemployed: Systematic Review
Source: JMIR Public Health Surveill. 2025 May 5;11:e65698. doi: 10.2196/65698 (PMC12089865; doi:10.2196/65698)
Supplement: Multimedia Appendix 5 [file publichealth_v11i1e65698_app5.docx]

**Multimedia Appendix 4.** Overview of the measurement instruments used.

| Instruments | Syndromal |  |
| --- | --- | --- |
|  | *Depression severity* | Kessler K-10 (*n* = 1)  HSCL (*n* = 6)  PHQ – 9 (*n* = 1) |
|  | *Anxiety and depression* | HADS (*n* =1)  DASS (*n* = 1)  GHQ - 30 (*n* = 1)  PHQ -D (*n* =1) |
|  |  |  |
|  | Sub-Syndromal |  |
|  | *Affect*  *Mental Health* | PANAS (*n* = 1)  SF-36 Mental health composite (*n* = 2)  KÖPS (*n* = 1) |

*Note.* DASS: Depression Anxiety Stress Scale; GHQ: General Health Questionnaire; HADS: Hospital Anxiety and Depression Scale; HSCL: Hopkins Symptoms Checklist; Kessler K-10: Kessler K-10 Screening Scale for Psychological Distress; KÖPS: Questionnaire on Physical, Psychological and Social Symptoms (Fragebogen für körperliche, psychische und soziale Symptome); PANAS: Positive and Negative Affect Scale; PHQ - 9: Patient Health Questionnaire; PHQ - D: German validation of the Patient Health Questionnaire; SF-36
