# Supplementary material for: Interventions to Foster Mental Health and Reintegration in Individuals Who Are Unemployed: Systematic Review
Source: JMIR Public Health Surveill. 2025 May 5;11:e65698. doi: 10.2196/65698 (PMC12089865; doi:10.2196/65698)
Supplement: Multimedia Appendix 6 [file publichealth_v11i1e65698_app6.docx]

**Multimedia Appendix 5.** Detailed overview of all ratings and judgements.

| **Non randomized trials** | | | | | | | | | | |
| --- | --- | --- | --- | --- | --- | --- | --- | --- | --- | --- |
| **Barry et al., 2006** | | | | | | | | | | |
| **Outcome(s) observed** | **Bias domain** | | | **Outcome assessed (separately/grouped)** | | | **Authors Judgement of Quality** | | | **Reasons for assessment** |
| O_1_: depressive symptoms  O_2_: Re employment | Bias due to confounding** | | | Grouped (O_1_, O_2_): | | | Moderate risk | | | - Significant baseline differences regarding gender and length of unemployment, regression used to control for confounders |
|  | Bias in selection of participants | | | Grouped (O_1_, O_2_): | | | Serious | | | - Selection of participants carried out by training agencies |
|  | Bias in classification of interventions | | | Grouped (O_1_, O_2_): | | | Low | | | - Clear allocation to intervention and control groups |
|  | Bias due to deviation from intended interventions | | | Grouped (O_1_, O_2_): | | | Low | | | - Apparently, no deviations and group changers in the intervention phase |
|  | Bias due to missing data*** | | | Grouped (O_1_, O_2_): | | | NI | | | - No information obtainable |
|  | Bias in measurement of outcomes | | | Grouped (O_1_, O_2_): | | | NI | | | - No information obtainable |
| **Carlier et al., 2018** | | | | | | | | | | |
| **Outcome(s) observed** | **Bias domain** | | | **Outcome assessed (separately/grouped)** | | | **Authors Judgement of Quality** | | | **Reasons for assessment** |
| O_1_: Anxiety and depressive Symptoms (Kessler K10)  O_2_: participation in paid employment | Bias due to confounding* | | | Grouped (O_1_, O_2_): | | | Moderate risk | | | - Mean age and length of last employment differed between groups - Appropriate use of propensity score to control for confounders |
|  | Bias in selection of participants | | | Grouped (O_1_, O_2_): | | | Critical risk | | | - Persons with common mental health problems were preferably referred to the interdisciplinary re-employment program |
|  | Bias in classification of interventions | | | Grouped (O_1_, O_2_): | | | Low | | | - Interventions are actively assigned by the researchers |
|  | Bias due to deviation from intended interventions | | | Grouped (O_1_, O_2_): | | | Low | | | - Participants did adhere to the assigned interventions, no crossovers between intervention groups reported |
|  | Bias due to missing data** | | | Grouped (O_1_, O_2_): | | | NI | | | - Markov chain Monte Carlo (MCMC) method was used, but no information on the amount of complete cases given |
|  | Bias in measurement of outcomes | | | O_1_:  O_2_ : | | | Moderate risk  Low risk | | | - Method of outcome assessment (Kessler K10) subjectively assessed, knowledge of intervention received possibly influenced outcome measure - Objective measure used (Participation in paid employment) |
|  | Bias in selection of the reported results | | | Grouped (O_1_, O_2_): | | | Moderate | | | - Protocol? Reported results correspond to intended outcomes, and are internally and externally consistent. No indication of selection of the reported analysis |
| **Hulshof et al., 2020** | | | | | | | | | | |
| **Outcome(s) observed** | **Bias domain** | | | **Outcome assessed (separately/grouped)** | | | **Authors Judgement of Quality** | | | **Reasons for assessment** |
| O_1_: Affect (PANAS)  O_2_: Re employment | Bias due to confounding* | | | Grouped (O_1_, O_2_): | | | Low | | | - Groups matched based on gender and duration of unemployment |
|  | Bias in selection of participants | | | Grouped (O_1_, O_2_): | | | Low | | | - High number of possible participants (N = 1308) invited via E-Mail and by letters via the agency |
|  | Bias in classification of interventions | | | Grouped (O_1_, O_2_): | | | Low | | | - Intervention status is well defined |
|  | Bias due to deviation from intended interventions | | | Grouped (O_1_, O_2_): | | | Low | | | - Apparently, no deviations and group changers in the intervention phase |
|  | Bias due to missing data** | | | Grouped (O_1_, O_2_): | | | Moderate risk | | | - Proportions of missing outcomes differed slightly across intervention groups, dropout pattern completely random |
|  | Bias in measurement of outcomes | | | O_1_:  O_2_ : | | | Low  Low | | | - Validated instrument used - Reemployment status provided by the agency |
|  | Bias in selection of the reported results | | | Grouped (O_1_, O_2_): | | | Low | | | - No indication of selection of the reported results |
| **Reynolds et al., 2010** | | | | | | | | | | |
|  | **Bias domain** | | |  | | | **Authors Judgement of Quality** | | | **Reasons for assessment** |
| O_1_: Depressive symptoms (HSCL)  O_2_: Re employment | Bias due to confounding* | | | Grouped (O_1_, O_2_) | | | Moderate risk | | | - Significant baseline differences regarding gender and length of unemployment, regression used to control for confounders |
|  | Bias in selection of participants | | | Grouped (O_1_, O_2_) | | | Serious risk | | | - Selection of the participants done by agencies, focusing on those regarded as difficult to place for inclusion in the intervention group |
|  | Bias in classification of interventions | | | Grouped (O_1_, O_2_) | | | Low | | | - Intervention status is well defined |
|  | Bias due to deviation from intended interventions | | | Grouped (O_1_, O_2_) | | | Low | | | - Any deviation from the intervention reflected usual practice |
|  | Bias due to missing data** | | | Grouped (O_1_, O_2_) | | | Low | | | - 93.1% of the data from the intervention group and 190 (95.96%) from the comparison group were valid and used for data analysis. |
|  | Bias in measurement of outcomes | | | O_1_  O_2_ | | | Low  NI | | | - Validated instrument was used - No information obtainable |
|  | Bias in selection of the reported results | | |  | | |  | | | - No information obtainable |
| **Rose et al., 2001** | | | | | | | | | | |
| **Outcome(s) observed** | | **Bias domain** | | | **Outcome assessed (separately/grouped)** | | | **Authors Judgement of Quality** | | **Reasons for assessment** |
| O_1_: Mental health (SF-36 Mental Health Composite)  O_2_: Reemployment | | Bias due to confounding* | | | Grouped (O_1_, O_2_): | | | Low | | - No significant baseline differences among confounding variables |
|  | | Bias in selection of participants | | | Grouped (O_1_, O_2_): | | | Moderate | | - study had difficulty maintaining recruitment through institutional structures thus impacting randomization into groups |
|  | | Bias in classification of interventions | | | Grouped (O_1_, O_2_): | | | Low | | - Intervention status is well defined |
|  | | Bias due to deviation from intended interventions | | | Grouped (O_1_, O_2_): | | | Low | | - Apparently, no deviations and group changers in the intervention phase |
|  | | Bias due to missing data** | | | Grouped (O_1_, O_2_): | | | Serious risk | | - Proportions of missing participants differ substantially across groups (43.5% in IG, 18.7% in CG) |
|  | | Bias in measurement of outcomes | | | O_1_:  O_2_ : | | | Low  Moderate risk | | - Validated instrument used - Re-employment assessed via self-rating |
|  | | Bias in selection of the reported results | | | Grouped (O_1_, O_2_): | | |  | | - No indication of selection of the reported results |
| **Rothländer et al., 2012** | | | | | | | | | | |
|  | **Bias domain** | |  | | | **Authors Judgement of Quality** | | | **Reasons for assessment** | |
| O_1_: Mental health problems (KÖPS-psychisch)  O_2_: Re employment (Benefit receipt status) | Bias due to confounding* | | Grouped (O_1_, O_2_) | | | Low risk | | | - No significant baseline differences among confounding variables | |
|  | Bias in selection of participants | | Grouped (O_1_, O_2_) | | | Low | | | - A systematic selection bias by employment providers seems unlikely due to heterogeneity of the groups | |
|  | Bias in classification of interventions | | Grouped (O_1_, O_2_) | | | Low | | | - Clear allocation to intervention and control groups | |
|  | Bias due to deviation from intended interventions | | Grouped (O_1_, O_2_) | | | Low | | | - Apparently, no deviations and group changers in the intervention phase | |
|  | Bias due to missing data** | | Grouped (O_1_, O_2_) | | | Serious | | | - Proportions of and reasons for missing participants differ substantially across intervention groups | |
|  | Bias in measurement of outcomes | | O_1_  O_2_ | | | Low  Moderate | | | - Validated instruments were used - change in benefit receipt status recorded within the study period. but unclear how this was implemented | |
|  | Bias in selection of the reported results | |  | | |  | | | - no information | |

**Randomized-Controlled Trials**

| **Caplan et al., 1989** | | | | |
| --- | --- | --- | --- | --- |
|  | **Bias domain** |  | **Authors Judgement of Quality** | **Reasons for assessment** |
| O_1_: Mental Health (HSC)  O_2_: Re employment | Randomization process | Grouped (O_1_, O_2_) | Some concerns | - only information "randomly assigned" given |
|  | Deviations from intended intervention (adherence) | Grouped (O_1_, O_2_) | Some concerns | - Participants and personal aware of assignment during trial |
|  | Missing outcome | Grouped (O_1_, O_2_) | Low | - High rates of respondents rates over time of survey, experimental analyses based on subset of complete cases |
|  | Measurement | O_1_  O2 | Low  Low | - Appropriate measurement, validated scale - Re employment specifically defined (working at least 20h/week and characterized as “working enough”) |
|  | Result selection | Grouped (O_1_, O_2_) | Some concerns | - No information on pre-specification of outcomes |
| **Della-Posta et al., 2006** | | | | |
|  | **Bias domain** |  | **Authors Judgement of Quality** | **Reasons for assessment** |
| O_1_: Depression and Anxiety (DASS)  O_2_: Re employment | Randomization process | Grouped (O_1_, O_2_) | Some concerns | - only information "randomly assigned" given |
|  | Deviations from intended intervention (adherence) | Grouped (O_1_, O_2_) | Some concerns | - Participants and personal aware of assignment during trial |
|  | Missing outcome | Grouped (O_1_, O_2_) | Low | - Outcome data available for all participants |
|  | Measurement | O_1_  O2 | Low  Low | - Appropriate measurement, validated scale - Employment status assessed and verified independently by the rehabilitation provider*** |
|  | Result selection | Grouped (O_1_, O_2_) | Some concerns | - No protocol available*** |
| **Harris et al., 2002** | | | | |
|  | **Bias domain** |  | **Authors Judgement of Quality** | **Reasons for assessment** |
| O_1_: Mental health (SF-36; MCS)  O_2_: Employment status | Randomization process | Grouped (O_1_, O_2_) | Some concerns | - only information "allocated in random blocks” |
|  | Deviations from intended intervention (adherence) | Grouped (O_1_, O_2_) | Some concerns | - Participants and personal aware of assignment during trial |
|  | Missing outcome | Grouped (O_1_, O_2_) | Low | - Outcome data available for all participants |
|  | Measurement | O_1_  O2 | Low  NI | - Appropriate measurement, validated scale - No information obtainable |
|  | Result selection | Grouped (O_1_, O_2_) | Some concerns | - No information on pre-specification of outcomes |
| **Herbig et al., 2012** | | | | |
|  | **Bias domain** |  | **Authors Judgement of Quality** | **Reasons for assessment** |
| O_1_: Mental Health (PHQ-D)  O_2_: Re employment | Randomization process | Grouped (O_1_, O_2_) | Low | - Stratification process used for randomization |
|  | Deviations from intended intervention (adherence) | Grouped (O_1_, O_2_) | Some concerns | - Participants and personal aware of assignment during trial |
|  | Missing outcome | Grouped (O_1_, O_2_) | Low | - Outcome data available for all participants |
|  | Measurement | O_1_  O_2_ | Low | - Appropriate measurements, validated scale used for mental health - Re-employment objectively assessed |
|  | Result selection | Grouped (O_1_, O_2_) | NI | - No information on pre-specification of outcomes |
| **Himle et al., 2012** | | | | |
|  | **Bias domain** |  | **Authors Judgement of Quality** | **Reasons for assessment** |
| O_1_: Depression (PHQ-9)  O_2_: Re employment (Hours worked per week) | Randomization process | Grouped (O_1_, O_2_) | Low | - Cohort randomization; opaque sealed envelopes used |
|  | Deviations from intended intervention (adherence) | Grouped (O_1_, O_2_) | Low | - Assessment personnel was blinded |
|  | Missing outcome | Grouped (O_1_, O_2_) | Low | - Appropriate statistical measure used for dealing with missing values |
|  | Measurement | O_1_  O_2_ | Low  Some concerns | - Appropriate measurements, validated scale used for mental health - Self-report measures for hours worked per week |
|  | Result selection | Grouped (O_1_, O_2_) | NI | - Pilot trial, results probably in accordance with pre-specified outcomes |
| **Maguire et al., 2014** | | | | |
|  | **Bias domain** |  | **Authors Judgement of Quality** | **Reasons for assessment** |
| O_1_: Anxiety and Depression (HADS)  O_2_: Re-employment | Randomization process | Grouped (O_1_, O_2_) | Some concerns | - “Individuals randomly allocated” |
|  | Deviations from intended intervention (adherence) | Grouped (O_1_, O_2_) | Some concerns | - Participants and personal aware of assignment during trial |
|  | Missing outcome | Grouped (O_1_, O_2_) | Some concerns | - Outcome data not available for all participants, appropriate statistical measures used to test for patterns of drop-out |
|  | Measurement | O_1_  O_2_ | Low  Some concerns | - Appropriate measurements, validated scale used for anxiety and depression - Self-report measures for hours worked per week |
|  | Result selection | Grouped (O_1_, O_2_) | Low | - Protocol available*** |
| **Proudfoot et al., 1997** | | | | |
|  | **Bias domain** |  | **Authors Judgement of Quality** | **Reasons for assessment** |
| O_1_: Mental strain (GHQ-30)  O_2_: Reemployment | Randomization process | Grouped (O_1_, O_2_) | Low | - “Allocation was generated by a random numbers table” |
|  | Deviations from intended intervention (adherence) | Grouped (O_1_, O_2_) | Low | - Investigators aware of group allocation, but were accompanied in by co-trainers who were non-investigators all programmes, participants were unaware of group allocation |
|  | Missing outcome | Grouped (O_1_, O_2_) | Low | - Analyses based on complete cases |
|  | Measurement | O_1_  O_2_ | Low  NI | - Appropriate measurements, validated scale used for anxiety and depression - No information on how reemployment was assessed |
|  | Result selection | Grouped (O_1_, O_2_) | Low | - Results probably in accordance with pre-specified outcomes |
| **Vinokur et al., 1995** | | | | |
|  | **Bias domain** |  | **Authors Judgement of Quality** | **Reasons for assessment** |
| O_1_: Depression (HSC)  O_2_: Re-employment | Randomization process | Grouped (O_1_, O_2_) | Low | - Computerized randomization procedure |
|  | Deviations from intended intervention (adherence) | Grouped (O_1_, O_2_) | NI | - Participants and personal likely to be aware of assignment during trial |
|  | Missing outcome | Grouped (O_1_, O_2_) | Low | - No significant difference in attrition between the groups |
|  | Measurement | O_1_  O_2_ | Low  Low | - Validated instrument used - Re employment specifically defined (reported number of paid work per week and number of months respondents reported having worked for at least 35hr/week |
|  | Result selection | Grouped (O_1_, O_2_) | NI | - No information of pre-specification of outcomes |
| **Vinokur et al., 2000** | | | | |
|  | **Bias domain** |  | **Authors Judgement of Quality** | **Reasons for assessment** |
| O_1_: Depression (HSC)  O_2_: Reemployment | Randomization process | Grouped (O_1_, O_2_) | Low | - Computerized randomization procedure |
|  | Deviations from intended intervention (adherence) | Grouped (O_1_, O_2_) | NI | - Participants and personal likely to be aware of assignment during trial |
|  | Missing outcome | Grouped (O_1_, O_2_) | Low | - No significant difference in attrition between the groups |
|  | Measurement | O_1_  O_2_ | Low  Low | - Validated instrument used - Re employment specifically defined (working at least 20h/week and characterized as “working enough”) |
|  | Result selection | Grouped (O_1_, O_2_) | NI | - No information of pre-specification of outcomes |
| **Vuori et al., 2002** | | | | |
|  | **Bias domain** |  | **Authors Judgement of Quality** | **Reasons for assessment** |
| O_1_: depressive symptoms (Finnish scale based on Hopkins checklist)  O_2_: Re-employment | Randomization process | Grouped (O_1_, O_2_) | Some | - Only information given that participants “were randomized” |
|  | Deviations from intended intervention (adherence) | Grouped (O_1_, O_2_) | NI | - Participants and personal likely to be aware of assignment during trial |
|  | Missing outcome | Grouped (O_1_, O_2_) | Low | - Appropriate statistical measure used for dealing with missing values |
|  | Measurement | O_1_  O_2_ | Low  Some concerns | - Validated instrument used - Employment status assessed by self-report |
|  | Result selection | Grouped (O_1_, O_2_) | NI | - No information of pre-specification of outcomes |
| **Vuori et al., 2005** | | | | |
|  | **Bias domain** |  | **Authors Judgement of Quality** | **Reasons for assessment** |
| O_1_: Depressive symptoms (Finnish scale based on Hopkins checklist)  O_2_: Re-employment | Randomization process | Grouped (O_1_, O_2_) | Some | - Only information given that participants “were randomized” |
|  | Deviations from intended intervention (adherence) | Grouped (O_1_, O_2_) | NI | - Participants and personal likely to be aware of assignment during trial |
|  | Missing outcome | Grouped (O_1_, O_2_) | Low | - T4 questionnaires returned by 90.6% |
|  | Measurement | O_1_  O_2_ | Low  Some concerns | - Validated instrument used - Employment status assessed by self-report |
|  | Result selection | Grouped (O_1_, O_2_) | NI | - No information of pre-specification of outcomes |

Please note that when using a different rating tool, the overall and domain-specific assessment may vary.

*confounders specified by the authors: age, gender, length of unemployment

**missing data defined as: data missing from complete data set after receiving the intervention (e.g. T1)

*** additional information was obtained by contacting the authors

Reference List:

Barry M, Reynolds C, Sheridan A, Egenton R. Implementation of the JOBS programme in Ireland. Journal of Public Mental Health. 2006.

Caplan RD, Vinokur AD, Price RH, van Ryn M. Job seeking, reemployment, and mental health: a randomized field experiment in coping with job loss. J Appl Psychol. 1989 Oct;74(5):759-69. PMID: 2793774. doi: <https://10.1037/0021-9010.74.5.759>.

Carlier BE, Schuring M, Burdorf A. Influence of an Interdisciplinary Re-employment Programme Among Unemployed Persons with Mental Health Problems on Health, Social Participation and Paid Employment. J Occup Rehabil. 2018 Mar;28(1):147-57. PMID: 28397017. doi: <https://10.1007/s10926-017-9704-3>.

Della-Posta C, Drummond PD. Cognitive behavioural therapy increases re-employment of job seeking worker's compensation clients. J Occup Rehabil. 2006 Jun;16(2):223-30. PMID: 16705491. doi: <https://10.1007/s10926-006-9024-5>.

Harris E, Lum J, Rose V, Morrow M, Comino E, Harris M. Are CBT interventions effective with disadvantaged job-seekers who are long-term unemployed? Psychology, Health & Medicine. 2002;7(4):401-10. PMID: 2002-06931-004. doi: <https://10.1080/1354850021000015221>.

Herbig B, Glaser J, Angerer P. Old, sick, unemployed, without a chance? Results of a randomised controlled trial of the effects of a combined health and employment promotion program for the older long-term unemployed (AmigA-M). Bundesgesundheitsblatt Gesundheitsforschung Gesundheitsschutz. 2012 Aug;55(8):970-9. PMID: 22842891. doi: <https://10.1007/s00103-012-1514-3>.

Himle JA, Bybee D, Steinberger E, Laviolette WT, Weaver A, Vlnka S, et al. Work-related CBT versus vocational services as usual for unemployed persons with social anxiety disorder: A randomized controlled pilot trial. Behav Res Ther. 2014 Dec;63:169-76. PMID: 25461793. doi: <https://10.1016/j.brat.2014.10.005>.

Hulshof IL, Demerouti E, Le Blanc PM. Providing Services During Times of Change: Can Employees Maintain Their Levels of Empowerment, Work Engagement and Service Quality Through a Job Crafting Intervention? Front Psychol. 2020;11:87. PMID: 32047468. doi: <https://10.3389/fpsyg.2020.00087>.

Maguire N, Hughes VC, Bell L, Bogosian A, Hepworth C. An evaluation of the choices for well-being project. Psychol Health Med. 2014;19(3):303-15. PMID: 23822617. doi: <https://10.1080/13548506.2013.806813>.

Proudfoot J, Guest D, Carson J, Dunn G, Gray J. Effect of cognitive-behavioural training on job-finding among long-term unemployed people. Lancet. 1997 Jul 12;350(9071):96-100. PMID: 9228961. doi: <https://10.1016/s0140-6736(96)09097-6>.

Reynolds C, Barry MM, Gabhainn SN. Evaluating the impact of the winning new jobs programme on the re-employment and mental health of a mixed profile of unemployed people. International Journal of Mental Health Promotion. 2010;12(2):32-41. PMID: 2010-10753-006. doi: <https://10.1080/14623730.2010.9721812>.

Rose V. Improving the Health of People who are Unemployed through the Job Network. A study of a brief CBT intervention in South Western Sydney. 2001.

Rothländer K, Mühlpfordt S, Richter P. Evaluation des Gesundheitsförderungsprogramms 'Aktive Bewältigung von Arbeitslosigkeit(AktivA)' = Evaluation of the health promotion program 'Active Coping with Unemployment (AktivA)'. Zeitschrift für Gesundheitspsychologie. 2012;20(3):115-27. PMID: 2012-19868-002. doi: <https://10.1026/0943-8149/a000070>.

Vinokur AD, Price RH, Schul Y. Impact of the JOBS intervention on unemployed workers varying in risk for depression. Am J Community Psychol. 1995 Feb;23(1):39-74. PMID: 7572826. doi: <https://10.1007/bf02506922>.

Vinokur AD, Schul Y, Vuori J, Price RH. Two years after a job loss: long-term impact of the JOBS program on reemployment and mental health. J Occup Health Psychol. 2000 Jan;5(1):32-47. PMID: 10658883. doi: <https://10.1037//1076-8998.5.1.32>.

Vuori J, Silvonen J. The benefits of a preventive job search program on re‐employment and mental health at 2‐year follow‐up. Journal of Occupational and Organizational Psychology. 2005;78(1):43-52. doi: <https://doi.org/10.1348/096317904X23790>.

Vuori J, Silvonen J, Vinokur AD, Price RH. The Työhön Job Search Program in Finland: benefits for the unemployed with risk of depression or discouragement. J Occup Health Psychol. 2002 Jan;7(1):5-19. PMID: 11827233. doi: https://10.1037//1076-8998.7.1.5.
